# Fibroblast growth factor 21 reflects liver fat accumulation and dysregulation of signalling pathways in the liver of C57BL/6J mice

**DOI:** 10.1038/srep30484

**Published:** 2016-07-29

**Authors:** Fenni Rusli, Joris Deelen, Evi Andriyani, Mark V. Boekschoten, Carolien Lute, Erik B. van den Akker, Michael Müller, Marian Beekman, Wilma T Steegenga

**Affiliations:** 1 Nutrition, Metabolism & Genomics Group, Division of Human Nutrition, Wageningen University, 6700 EV Wageningen, The Netherlands; 2 Department of Molecular Epidemiology, Leiden University Medical Center, Leiden, The Netherlands; 3 The Delft Bioinformatics Lab, Delft University of Technology, Mekelweg 4, 2628 CD, Delft, The Netherlands; 4 Norwich Medical School, University of East Anglia, Norwich, UK

## Abstract

Fibroblast growth factor 21 (Fgf21) has emerged as a potential plasma marker to diagnose non-alcoholic fatty liver disease (NAFLD). To study the molecular processes underlying the association of plasma Fgf21 with NAFLD, we explored the liver transcriptome data of a mild NAFLD model of aging C57BL/6J mice at 12, 24, and 28 months of age. The plasma Fgf21 level significantly correlated with intrahepatic triglyceride content. At the molecular level, elevated plasma Fgf21 levels were associated with dysregulated metabolic and cancer-related pathways. The up-regulated Fgf21 levels in NAFLD were implied to be a protective response against the NAFLD-induced adverse effects, e.g. lipotoxicity, oxidative stress and endoplasmic reticulum stress. An *in vivo* PPARα challenge demonstrated the dysregulation of PPARα signalling in the presence of NAFLD, which resulted in a stochastically increasing hepatic expression of *Fgf21*. Notably, elevated plasma Fgf21 was associated with declining expression of *Klb*, Fgf21’s crucial co-receptor, which suggests a resistance to Fgf21. Therefore, although liver fat accumulation is a benign stage of NAFLD, the elevated plasma Fgf21 likely indicated vulnerability to metabolic stressors that may contribute towards progression to end-stage NAFLD. In conclusion, plasma levels of Fgf21 reflect liver fat accumulation and dysregulation of metabolic pathways in the liver.

Non-alcoholic fatty liver disease (NAFLD) has been recognised as a hepatic manifestation of metabolic syndrome. NAFLD covers a spectrum of liver injuries ranging from fat accumulation in the liver (steatosis) to the more severe condition of steatohepatitis (NASH). Considering that NAFLD is currently the most common liver disorder, prevalence of which has been reported to be 20–40% in the US^1^,^2^, the population would benefit from a diagnosis from the early stage of NAFLD. Intervention as simple as weight management through diet and exercise is the most effective way leading to reduced liver fat, NASH remission, and also reduction of fibrosis^3^. However, at present a population-based screening tool for NAFLD is still lacking. Serum screening of liver enzymes and a liver ultrasound technique have been employed in screenings and clinical studies^4^, but these procedures are, for different reasons, suboptimal. A number of studies have pointed out that the blood-screening test of the commonly analysed liver enzymes, including alanine aminotransferase (ALT), aspartate aminotransferase (AST), alkaline phosphatase (ALP), γ-glutamyl-transpeptidase (γ-GT) and albumin, poorly diagnose NAFLD^5^,^6^,^7^,^8^. The ultrasound technique has better accuracy than the blood screening test, but is also suboptimal due to the low sensitivity of this method^9^. Magnetic resonance imaging and spectroscopy has a higher sensitivity, but this technique requires specific and expensive instruments, which limits its measurement availability. Diagnosis on liver biopsies is the most accurate way to determine the presence of NAFLD, but this procedure is highly invasive and not suitable for population-based screening. Hence, new accurate and non-invasive measures are required for the diagnosis of NAFLD.

Recently, fibroblast growth factor 21 (FGF21 (Fgf21 in mice)) has emerged as a potential diagnostic marker for NAFLD. Serum FGF21 is found to be elevated in NAFLD patients, as compared to healthy subjects, and correlates with hepatic fat content and the degree of liver steatosis^4^,^10^,^11^,^12^. Moreover, the performance of FGF21 has been examined in a 3-year prospective study in China and high serum FGF21 was found to be a determinant of NAFLD, showing an area under curve of receiver operating characteristic (AUROC) of 0.816^13^. Serum FGF21 has also been reported to increase the accuracy of non-alcoholic steatohepatitis (NASH) diagnosis using cytokeratin-18 fragment (CK-18)^14^. Therefore, plasma/serum FGF21 seems a promising diagnostic marker for an accurate and non-invasive diagnosis of NAFLD.

FGF21 has multiple metabolic functions, regulating energy homeostasis, glucose-lipid metabolism and insulin sensitivity^13^. However, it is currently unclear which metabolic functions of FGF21 underlie the association of plasma FGF21 level and NAFLD. In the present study, we aim to identify the putative molecular mechanisms that underlie the association of plasma FGF21 level with NAFLD. A complicating factor for a human study in this field is that the accurate assessment of NAFLD would require liver biopsies from healthy subjects, which is ethically undesirable. Therefore, we employed a cohort of aging mice to investigate the association between NAFLD, which was determined by IHTG level, and plasma Fgf21. The use of a cohort of aging mice, which consists of four different age time points, enables us to investigate whether plasma Fgf21 can act as a biomarker at different age time points. NAFLD has been reported in subjects of all ages, but particularly in middle to old age (40–65 years old in human)^15^, which indicates that NAFLD develops over many years. To create a mice cohort that simulates the slow onset of NAFLD, we included a medium-fat diet (MF; 25E% from fat) as a diet that induces the development of NAFLD. The energy contribution from fat in this diet group is considered mild, compared to other NAFLD-inducing dietary interventions that commonly acutely stimulate NAFLD pathologies within several weeks of feeding by applying a high-fat diet (45E% from fat)^16^,^17^. In addition to the MF diet, we introduced a normal diet (10E% from fat) as the control (C) group and a calorie restriction diet (CR; 30E% reduced feeding compared to control group) as a diet regimen that prevents NAFLD. Then, we investigated whether the use of plasma Fgf21 as marker for NAFLD in our mice cohort is comparable to what has previously been reported for humans. We next searched for the biological processes underlying the association of plasma Fgf21 with NAFLD by performing microarray analysis on liver mRNA by using two approaches. First, we searched for pathways and upstream regulators associated with plasma Fgf21 by performing a gene co-expression network analysis using weighted gene co-expression network analysis (WGCNA)^18^. In the second approach, we investigated the major transcriptional difference in NAFLD by performing gene set enrichment analysis (GSEA), and then determining the relevance of Fgf21 in the NAFLD-related pathways. To further investigate PPARα signalling as one of the dysregulated pathways, we examined the PPARα response to its agonist at the gene expression level. Since we found that some of the mice of 6 months of age displayed a high level of plasma Fgf21 in the absence of elevated IHTG levels, we also explored the microarray data to search for the functions of the genes associated with the elevated Fgf21 plasma level without accumulation of IHTG.

## Materials and Methods

### Ethics statement

The animal experiment was approved by the Local Committee for Care and Use of Laboratory Animals at Wageningen University (code number: drs-2010151b) and performed in accordance with the institutional and national guidelines for the care and use of animals.

### Mice aging study

The mice aging study was a part of the IDEAL mice aging cohort that has been described in detail previously^19^,^20^. Briefly, male C57BL/6J mice (age of 7 weeks) were purchased from Janvier (Cedex, France) and were housed in pairs of two in the light and temperature (20 °C)-controlled animal facility of Wageningen University (12-hour light/dark cycle, light on at 04.00). The mice were acclimated for 2 weeks, receiving standard AIN-93G (Research Diet Services, Wijk bij Duurstede, The Netherlands) upon arrival. All mice were provided with *ad libitum* access to water. The study design is presented in Supplementary Fig. S1. The diet intervention started at the age of 9 weeks. The mice were housed individually and randomly distributed into three intervention groups: 1) control diet (C, 10E% fat, n = 89) receiving AIN-93W diet *ad libitum*; 2) calorie restricted diet (CR, n = 117) receiving AIN-93W-CR in portions containing 70E% of the mean energy intake compared to the mice on the control diet; 3) medium fat diet (MF, 25E% fat, n = 127) receiving AIN-93W-MF *ad libitum*. AIN-93W-CR contains an increased concentration of vitamins and minerals in order to feed these mice the same concentrations of micronutrients as the mice receiving the AIN-93W diet and to avoid malnutrition. Portion sizes for the mice on the CR were based on food intake of mice on the control diet and adjusted every 6 months. The rations were provided each day at 15.30, 30 minutes before the light was switched off. The complete diet compositions are listed in Supplementary Table S1 (Research Diet Services, Wijk bij Duurstede, The Netherlands).

The mice were culled at the age of 6, 12, 24 and 28 months. At each sacrifice, 12–18 mice of each intervention group were sacrificed between 14.00–17.00 on consecutive days (the remaining mice stayed in the experiment and were evaluated at older ages). Mice were paired per dietary intervention group according to body weight at sacrifice, so that mock and PPARα agonist Wy-14,643 (Wy) treatment were provided to mice with similar body weight. Prior to sacrifice each mouse was first fasted for 4 hours, after which they received an intragastric gavage of either solvent (0.5% carboxymethyl cellulose) or Wy dispersed in solvent (160 mg Wy/kg body weight) and were fasted again for another 6 hours. Body weight, liver weight, IHTG and 4-hydroxyproline were measured in both the mock- and Wy-treated animals. Plasma Fgf21 and liver microarray analysis were performed for mock-treated animals only, as Wy substance is a PPARα agonist potentially affecting *Fgf21* expression. At the sacrifice, the mice were sedated with isoflurane (1.5%) in a mixture of nitrous oxide (70%) and oxygen (30%). Blood samples were collected by cardiac puncture, which was followed by neck dislocation. Weight of various organs was measured and, subsequently, organs/tissues were snap-frozen and stored at −80 °C until further molecular/biochemical analysis.

### Measurement of hepatic steatosis and fibrosis

Intrahepatic triglyceride (IHTG) content was determined in 5% liver homogenates prepared in buffer containing 250 mM sucrose, 1 mM EDTA, 10 mM Tris-HCl (pH 7.5), using the triglyceride Liquicolor Monoreagent (Instruchemie, Delfzijl, The Netherlands), according to manufacturer’s instruction. The IHTG level was applied as the diagnosis standard of NAFLD using the 5% or 50 mg TG per gram liver criterion from Kleiner’s scoring^21^. Liver fibrosis is represented by 4-hydroxyproline content measurement in the liver. The 4-hydroxyproline analysis was performed as previously described in Hillebrandt *et al*.^22^.

### Measurement of plasma Fgf21 level and other plasma markers

The plasma Fgf21 concentration was determined using Rat/Mouse FGF-21 ELISA kits (Milipore, cat #EZRMFGF21-26K), according to the manufacturer’s instructions. Plasma insulin was measured using a Mouse Adipokine (MADKMAG-71K) kit (Millipore, Billerica, MA, USA), according to the manufacturer’s instructions. The cytokeratin-18 plasma concentration was measured using Mouse Cytokeratin 18-M30 ELISA kit (Cusabio, Hubei, China).

### RNA isolation

Total RNA from liver*, tibialis anterior* muscle, colon scrapings and epidydimal white adipose tissue were isolated using TRIzol reagent (Invitrogen, Breda, The Netherlands), according to the manufacturer’s instructions. The RNA was treated with DNAse and purified on columns using the RNeasy microkit (Qiagen, Venlo, The Netherlands). RNA concentration was measured on a NanoDrop ND-1000 UV–vis spectrophotometer (Isogen, Maarsen, The Netherlands) and RNA integrity was checked on an Agilent 2100 Bioanalyzer (Agilent Technologies, Amsterdam, The Netherlands) with 6000 Nano Chips, according to the manufacturer’s instructions. RNA was judged as suitable only if samples showed intact bands of 18S and 28S ribosomal RNA subunits, displayed no chromosomal peaks or RNA degradation products and had a RNA integrity number (RIN) above 8.0.

### Microarray hybridization

Hybridization, washing and scanning of Affymetrix GeneChip Mouse Gene 1.1 ST arrays were performed according to standard Affymetrix protocols as described previously^19^,^23^. Microarray analysis was performed in MADMAX, a pipeline for statistical analysis of microarray data^24^. Arrays were normalized using the Robust Multiarray Average method^25^,^26^. Probe sets were defined according to Dai *et al*.^27^. In this method probes are assigned to unique gene identifiers, in this case Entrez IDs. The probes on the Gene 1.1 ST arrays represent 21,225 Entrez IDs. For the analysis, only genes having 1) an inter-quartile range of >0.1 and 2) an intensity value of >20 on at least five arrays were taken into account, which resulted in 15,885 genes in the dataset. Array data have been submitted to the Gene Expression Omnibus, with accession number GSE84495.

### cDNA synthesis and real-time quantitative PCR

The microarray data was validated by real-time quantitative PCR (Q-PCR). For each individual sample, single-stranded complementary DNA was synthesized from 1 μg of total RNA using the First Strand cDNA Synthesis kit (Thermo Scientific, Landsmeer, The Netherlands), following the supplier’s protocol. Q-PCR was performed using SensiMix SYBR No-ROX kit (Bioline, Alphen aan de Rijn, The Netherlands) and a CFX384 thermal cycler (Bio-Rad, Veenendaal, The Netherlands). The following thermal cycling conditions were used: 2 min at 94 °C, followed by 40 cycles of 94 °C for 15 s and 60 °C for 45 s. PCR reactions to validate *Fgf21* expression were performed in duplicate and all samples were normalized to *Rplp0* expression. Primer sequences were retrieved from the online PrimerBank database^28^ and the sequences of the primers used are listed in Supplementary Table S2.

### Statistical analysis

Data were analysed with GraphPad Prism 5.04. The data were expressed as mean ± standard error mean. Comparison between two groups was performed using student t-test, whereas comparison between 3 or more groups was performed using ANOVA. Correlation between two parameters was presented as Pearson correlation coefficient (r) and *p*-value. A *p*-value of <0.05 was considered significant. The receiver operating characteristic (ROC) curve analysis was carried out and the area under the ROC curves (AUROCs) were calculated to represent their performance to predict NAFLD. Optimal cut-off points were calculated for sensitivity and specificity reference (Youden Index).

### Hepatic transcriptomics data analysis

For the microarray data analysis, differentially expressed probe sets were identified by using linear models (library limma) and the intensity-based moderated t-statistic (IBMT) method was applied^29^,^30^. Resulting log_2_ intensities and *p*-values were used for further descriptive bioinformatic analysis of the data. Gene set enrichment analysis (GSEA; http://www.broad.mit.edu/gsea/) was performed in MADMAX^24^. Gene sets with a false discovery rate (FDR) q-value of <0.01 were considered significantly enriched.

Gene co-expression networks (modules) were constructed using the blockwiseModules R function in Weighted Gene Co-expression Network Analysis (WGCNA)^18^. WGCNA uses a network distance coupled with hierarchical clustering and dynamic tree cutting to define modules as branches of a cluster tree. Gene modules, which summarize the main patterns of variation, are defined in an unbiased fashion and denoted by colors. The first principal component represents the summary of the module and is referred to as the module eigengene (ME). MEs were then related to plasma Fgf21 level and other NAFLD-related traits. This approach avoids the multiple testing from thousands of individual transcripts to only a number of modules. To explore the functional pathways and predicted upstream regulators of the gene modules, Ingenuity pathway analysis (IPA; Ingenuity^®^ Systems) was used.

## Results

### Control and medium-fat diet groups developed NAFLD at middle and old age

**Figure 1:**
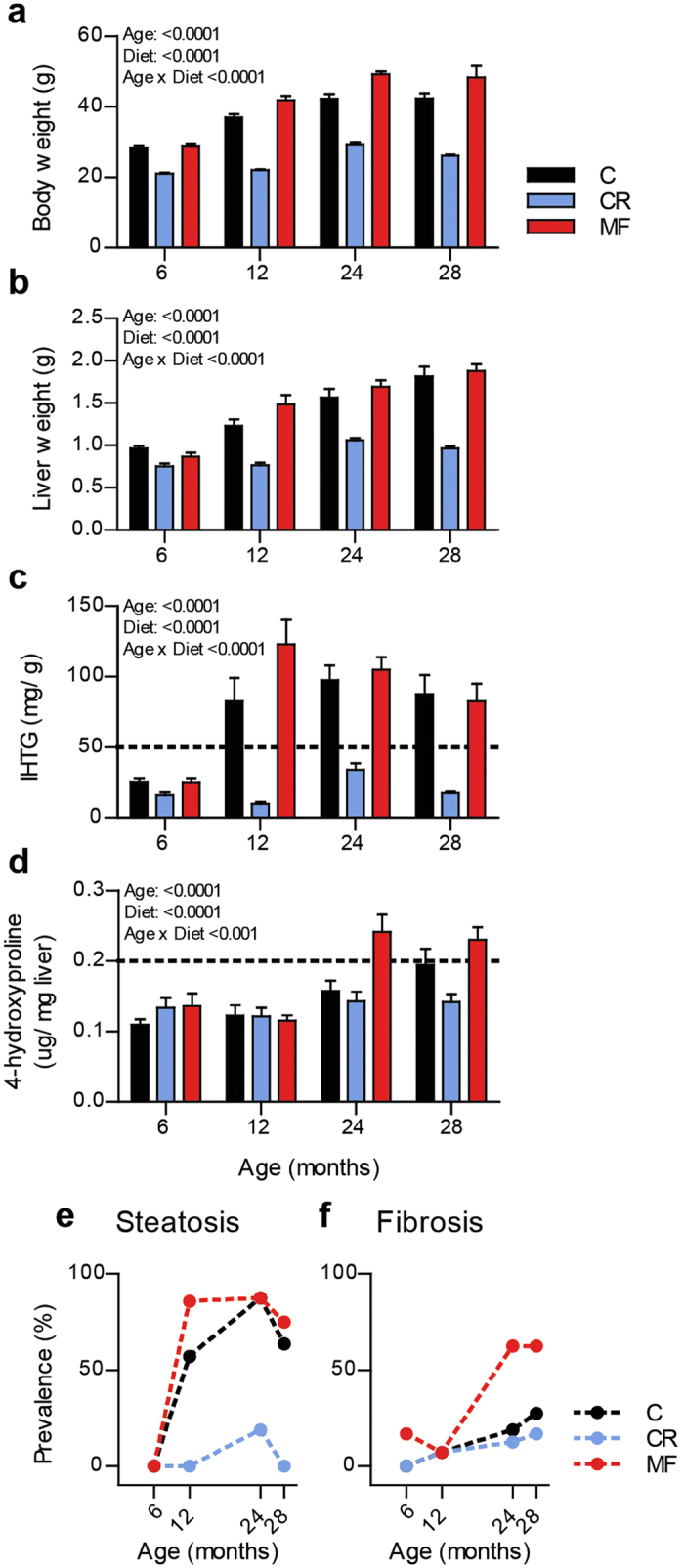
Physiological changes induced by the different dietary interventions at the age of 6, 12, 24, and 28 months. Body weight (**a**) liver weight (**b**) and IHTG content (**c**) dramatically increased over time, except for the CR-fed animals which stayed lean. Significance (*p*-value) of age, diet and interaction were evaluated using two-way ANOVA. Error bars represent s.e.m. Prevalence of hepatic steatosis (**e**) and fibrosis (**f**) in the mice aging cohort at different age time points.

The dietary interventions exerted pronounced effects on the mice’s body and liver weight, as shown in Fig. 1a,b, respectively. During aging, the MF-exposed animals gained the highest body and liver weight, while the CR-fed animals were the leanest. The weight gain was accompanied by an increase in IHTG content (Fig. 1c). While the CR-fed animals only showed a modest increase over time, the C- and MF-fed animals displayed an elevated IHTG level starting at middle-age, i.e. at 12 months. Liver fibrosis, which was represented by the measurement of 4-hydroxyproline (4-HP) content in the liver (Fig. 1d), showed to increase at old age (24 and 28 month) in the MF diet group.

The IHTG level was applied as the diagnosis standard of hepatic steatosis using the 5% or 50 mg TG per gram liver criterion from Kleiner’s scoring^21^. The prevalence of hepatic steatosis development in the cohort is depicted in Fig. 1e. At the age of 6 months, none of the mice in any of the diet groups developed hepatic steatosis, indicating a healthy liver condition at young/mature adult age. Hepatic steatosis development became visible in 12-month old mice exposed to C and MF diets, reaching up to 85.7% in the MF diet group. At the 24-month time point, the prevalence of hepatic steatosis increased considerably in the control group. At this age over 80% in the C and MF diet groups displayed steatosis (Fig. 1e) but at the age of 28 months, a slight decrease of prevalence was observed.

Since a criterion of 4-HP level for hepatic fibrosis diagnosis has not been clearly defined, we adapted the level of 4-HP that was reported by Fuchs *et al*. to be associated with extensive portal fibrosis (equivalent with Ishak fibrosis scoring stage 2–3)^31^. A level of >0.200 μg of 4-HP per mg liver was applied as an indication of liver fibrosis. Increasing prevalence of hepatic fibrosis was only pronounced in the MF intervention group at 24 and 28 months of age (Fig. 1f). These results showed that hepatic steatosis occurred in both the C and MF intervention groups at the middle-age time point, while hepatic fibrosis in particularly developed in the MF diet group at an old age. On the other hand, the CR-fed animals were protected from developing hepatic steatosis and fibrosis.

### Plasma Fgf21 reflected the hepatic fat accumulation at middle and old age, but not at mature adult age

**Figure 2:**
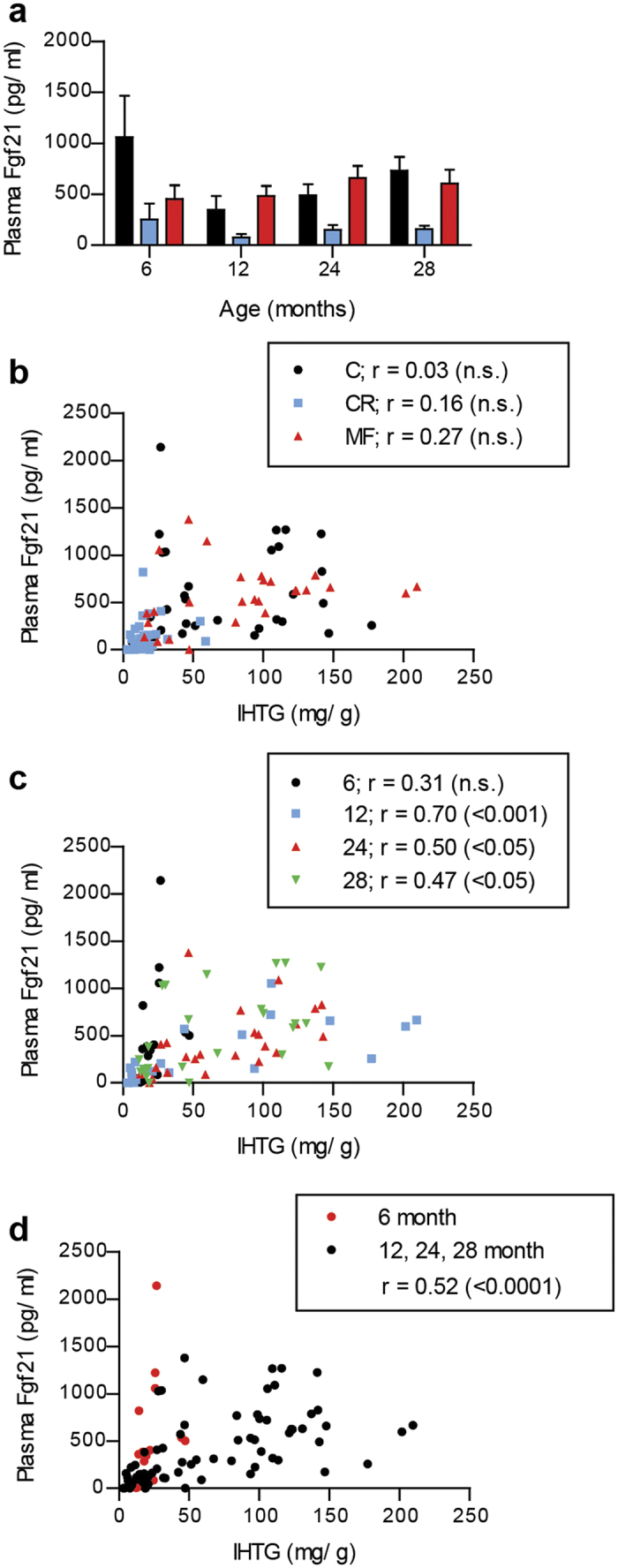
Plasma Fgf21 reflected the hepatic fat accumulation at middle and old age, but not at mature adult age. (**a**) Different plasma Fgf21 levels induced by the dietary interventions at the age of 6, 12, 24, and 28 months. Correlation of IHTG and plasma Fgf21 for different dietary interventions (**b**) and ages (**c**). (**d**) Plasma Fgf21 was positively correlated with IHTG levels at older ages, while the young 6-month-old mice had elevated plasma Fgf21, despite their low IHTG levels (in red symbols). r values were calculated with Pearson’s correlations and their significance are indicated in the parentheses.

The results presented in Fig. 2a show that plasma Fgf21 levels of the C- and MF-exposed animals were higher than that of the CR-exposed animals at all ages. Noticeably, plasma Fgf21 levels were particularly high at 6 months of age in the C and CR diet groups. Next, to examine a specific diet- or age-related effect on plasma Fgf21 levels, the correlation with IHTG content was carried out separately by diet and age. The CR diet group displayed low IHTG and plasma Fgf21 concentration, while the C and MF groups showed some variation. However, overall we did not observe significant correlation in any of the different diet groups (Fig. 2b). On the other hand, the comparison by age indicated that, except for the 6-month-old mice, the correlations at different ages were comparable (Fig. 2c). Some of the young mice, independent of their diet types, exhibited high plasma Fgf21 concentrations despite their low IHTG levels. This observation implies that the plasma Fgf21 concentration reflects the hepatic fat accumulation at middle-age (12 months) and old age (24 and 28 months), which results in an overall correlation coefficient of 0.52 (p < 0.0001), but performs differently at younger age (Fig. 2d).

### Plasma Fgf21 levels reflected liver *Fgf21* expression

**Figure 3:**
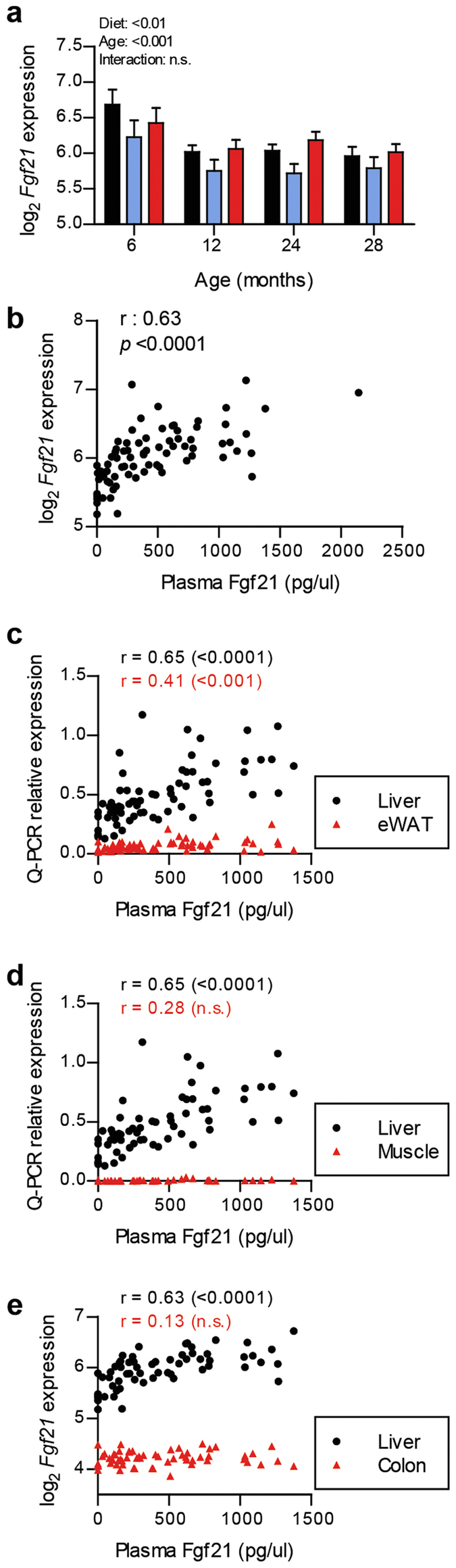
Plasma Fgf21 levels were strongly reflected by the expression of *Fgf21* in the liver. (**a**) Different expression levels of Fgf21 in the liver induced by the dietary interventions and age. (**b**) Significant positive correlation between plasma Fgf21 and *Fgf21* expression in the liver. The association between plasma and expression levels of Fgf21 was also compared in other tissue types, (**c**) epidydimal white adipose tissue (eWAT), (**d**) muscle, and (**e**) colon tissue. r values were calculated with Pearson’s correlations and their significance are indicated in the parentheses.

The gene expression levels of *Fgf21* in the liver were obtained from the microarray data and revealed that, similar to the plasma Fgf21 levels, the C- and MF-exposed animals displayed higher expression levels of *Fgf21* (Fig. 3a). Notably, the higher levels of plasma Fgf21 at the earliest time point of 6 months were also reflected in the *Fgf21* liver expression level. The results presented in Fig. 3b show that the plasma Fgf21 levels significantly correlated to hepatic *Fgf21* expression levels obtained by microarray analysis (r = 0.63, *p* < 0.0001). This result was confirmed by Q-PCR analysis (r = 0.71, *p* < 0.0001, Supplementary Fig. S2a). These observations indicate that the plasma Fgf21 concentration reflects the *Fgf21* mRNA levels in liver tissue. In addition, we examined whether plasma Fgf21 increased with body or liver weight. Correlation analyses confirmed that plasma Fgf21 had significant positive correlation with body and liver weights (Supplementary Fig. S2b,c). As Fgf21 has been reported to be expressed abundantly, not only in the liver, but also in other tissue types, we also examined whether the plasma Fgf21 concentrations reflect the *Fgf21* expression levels in epidydimal white adipose tissue (eWAT), *tibialis anterior* muscle and colon tissue. The results presented in Fig. 3c–e show that plasma Fgf21 levels was strongly associated with the liver expression, compared to the other tissues. The expression levels of *Fgf21* in the muscle and colon were extremely low (Fig. 3d,e). The correlation of plasma Fgf21 and expression in eWAT was significant, however, the levels of expression in the eWAT were much lower compared to those in the liver tissue (Fig. 3c).

To examine whether plasma Fgf21 in mice indicates hepatic fat accumulation as observed in humans, the mice were divided into two groups: those that either developed or did not develop NAFLD according to the IHTG criterion of higher or lower than 50 mg TG per gram liver from Kleiner’s scoring^21^. This resulted in 53 animals without NAFLD, which largely consisted of young or CR-fed animals, and 36 animals with NAFLD, which mostly consisted of older animals or under the C or MF dietary regimen. The average IHTG contents of the animals with and without NAFLD were 111.1 and 22.2 mg TG per gram liver, respectively. The characteristics of animals with and without NAFLD are summarized in Supplementary Table S3, showing that the animals with NAFLD had a significantly heavier body weight, larger epididymal fat depot, enlarged liver, and lower liver 4-HP content. The levels of fasting plasma insulin and the liver injury marker alanine aminotransferase (ALT) were elevated in the group of animals with NAFLD.

Subsequently, AUROC analysis was performed using optimal cut-off points determined by the Youden Index (plasma Fgf21 >222.0 pg/ml). The results summarized in Table 1 show that the sensitivity was remarkably high at 91.4%, while the specificity was low (57.1%). This resulted in an AUROC of 0.77 (Supplementary Fig. S3a) and an accuracy of 71.4% (positive predictive value/PPV of 60.4% and negative predictive value/NPV of 90.3%). Since plasma Fgf21 appears to perform differently at the age of 6 months and a previous study of obese children indicated that plasma Fgf21 did not provide additional value in predicting NAFLD^32^, we also tested the Fgf21 performance in older animals only. When the 6 months old animals were excluded, the AUROC and specificity of Fgf21 were improved to 0.84 (Supplementary Fig. S3b) and 73.5%, while the sensitivity remained high (88.6%). In addition, the accuracy was improved to 81.2% (PPV of 77.5% and NPV of 86.2%). Diagnosis using more markers commonly yield a better outcome, so we applied combinations of plasma Fgf21 with other plasma or trait markers for NAFLD, i.e. plasma ALT, plasma CK-18 and body weight. The performance accuracy described in Table 2 revealed that the plasma Fgf21 was best combined with plasma ALT (accuracy of 88.4%) or body weight (87.0%), while the combination with plasma CK-18 (81.2%) did not improve the performance of Fgf21.

### The gene module strongly correlated with the plasma Fgf21 levels contained steatosis- and cancer-related genes

To explore the molecular mechanism underlying the involvement of Fgf21 in NAFLD development, microarray analysis was performed on mRNA isolated from the livers of the 12, 24, and 28-month old animals. In the search for the molecular processes underlying the association of plasma Fgf21 with NAFLD, the microarray data were analysed by a two-step approach: 1) gene co-expression network analysis using WGCNA^18^ and 2) functional pathways and up-stream regulator analysis were determined by applying Ingenuity pathway analysis (IPA) on the co-expression network.

**Figure 4:**
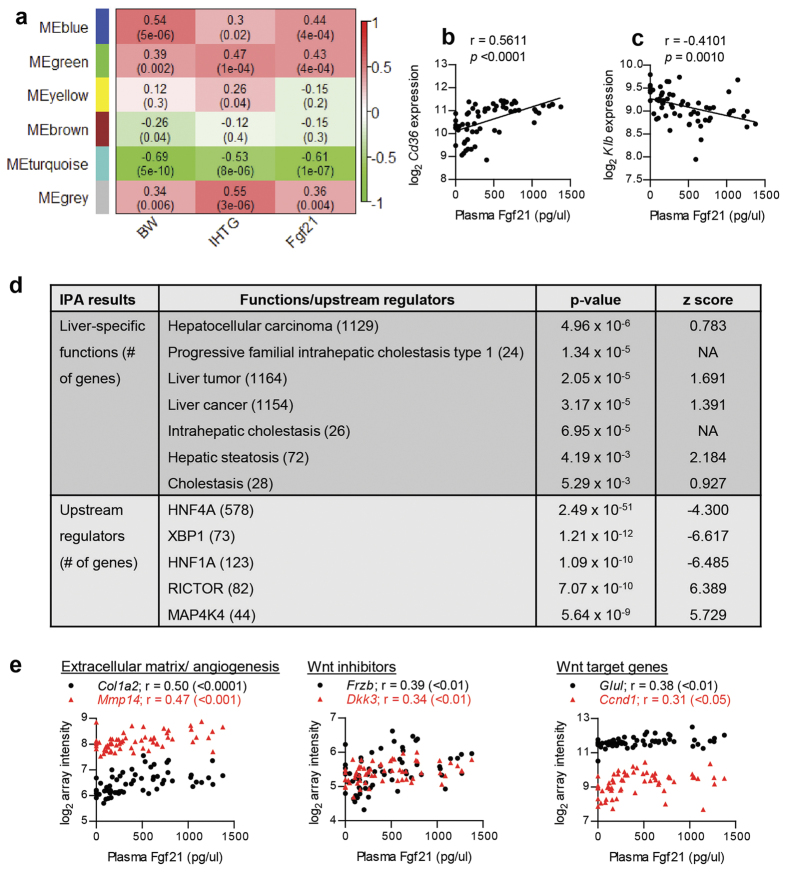
Liver biological processes associated with plasma Fgf21 level. (**a**) Heatmap depicting the correlation between gene modules (in rows) and phenotypes (in columns). The top values in each cell represents the correlation coefficient between the module and phenotype with the correlation *p*-value in parentheses. Red and green color represents positive and negative correlation, respectively. Correlation between plasma Fgf21 levels and hepatic expressions of *Cd36* (**b**) and *Klb* (**c**). (**d**) Biological processes and regulators associated with MEturquoise, which is the most significant modules correlated with plasma Fgf21. Significant liver-specific functions and upstream regulators are reported in *p*-values and z-scores. Positive and negative z-score represent predicted activation and inhibition, respectively. (**e**) Correlation between plasma Fgf21 and expression levels of hepatocellular carcinoma-related genes within MEturqouise, which included genes related to extracellular matrix formation and angiogenesis (*Col1a2* and *Mmp14*), inhibition of Wnt signalling (*Frzb* and *Dkk3*), and downstream target of Wnt signalling (*Glul* and *Ccnd1*). r values were calculated with Pearson’s correlations and their significance are indicated in the parentheses.

The WGCNA results presented in Fig. 4a, show that 5 modules were created from the hepatic transcriptome data and that 3 of them significantly correlated with plasma Fgf21 (*p* < 0.05; module grey denotes background genes outside of modules). The module displaying the most significant correlation with plasma Fgf21 was MEturquoise (r = −0.61). Interestingly, cluster differentiation 36 (*Cd36*), a fatty acid transporter gene involved in steatosis development, was identified as the top regulated gene in this module. A correlation analysis between plasma Fgf21 and *Cd36* expression levels showed a positive significant association (Fig. 4b). Intriguingly, beta-Klotho gene (*Klb*), a co-receptor component that is required for Fgf21 metabolic activity^33^,^34^, was included in this module. The expression of *Klb* decreased with the increase of plasma Fgf21 (Fig. 4c). In addition, strong inverse correlations with *Klb* expression were also observed for body weight and IHTG (Supplementary Fig. S4a). We also examined plasma Fgf21 correlation with the liver gene expression levels of the 4 members of the Fgf receptor family and observed a significant negative correlation with *Fgfr2* and *Fgfr4* expression, similarly to *Klb* (Supplementary Fig. S4b).

Next, we investigated which biological processes are represented by the genes in the MEturquoise module, by using IPA. Figure 4d shows the liver-specific functions with p-value < 0.01 and the 5 most significant regulators identified by IPA, which were ranked by p-value. Predicted activation/inhibition z-scores are displayed when available. The liver-specific functions associated with this module included hepatic steatosis and cholestasis, but the most significant function was the hepatocellular carcinoma (HCC). A number of genes related to HCC functions were identified in this module, including collagen type I, alpha 2 (*Col1a2*), matrix metallopeptidase 14 (*Mmp14*), frizzled-related protein (*Frzb*), dickkopf WNT signalling pathway inhibitor 3 (*Dkk3*), glutamate-ammonia ligase (*Glul*), and cyclin D1 (*Ccnd1*). The functions of these genes include extracellular matrix formation/angiogenesis (*Col1a2* and *Mmp14*), inhibitors of Wnt signalling (*Frzb* and *Dkk3*), and regulation of Wnt target genes (*Glul* and *Ccnd1*). The expression levels of these genes were significantly correlated with plasma Fgf21 levels (Fig. 4e), demonstrating that plasma Fgf21 level is associated with HCC-related signalling. Moreover, the identification of predicted upstream regulators further confirmed the association of this module with hepatocellular carcinoma (Fig. 4d): predicted inhibition of hepatocyte nuclear factor 4α and 1α (HNF4A and HNF1A), both are tumor suppressor regulators, while rapamycin-insensitive companion of mTOR (RICTOR) and mitogen-activated protein 4 kinase 4 (MAP4K4), factors involved in cancer development, were activated. X-box binding protein (XBP1) was also identified as one of the top upstream regulators.

### The elevated plasma Fgf21 levels in young animals without accumulation of IHTG were related to the up-regulation of lipid metabolism by PPARα, PPARGC1α and PPARγ

To elucidate the functions of the genes associated with the elevated Fgf21 plasma level at young age without accumulation of IHTG, WGCNA analysis was performed with the inclusion of the 6-month-old animals. For this purpose, we searched for a gene module that was significantly associated with plasma Fgf21, but not with IHTG, and we found that module MEgreen (1286 genes) fulfilled this criterion (Supplementary Fig. S5a). This module has a significant correlation with Fgf21 (r = 0.44, *p* < 0.0001), but not with IHTG (r = 0.004, *p *= 1). To screen for the genes strongly correlated with the plasma Fgf21, we filtered for the genes with a correlation coefficient larger than 0.4. IPA of biological functions revealed that these genes play a role in lipid metabolism (top 3 functions/regulators are listed in Supplementary Fig. S5b) and IPA identified peroxisome proliferator-activated receptor α (PPARα), peroxisome proliferator-activated receptor γ coactivator 1-α (PPARGC1α), and PPARγ as the predicted upstream regulators.

### NRF2 and PPARα targets, pathways differentially up-regulated by NAFLD, were linked to Fgf21

**Figure 5:**
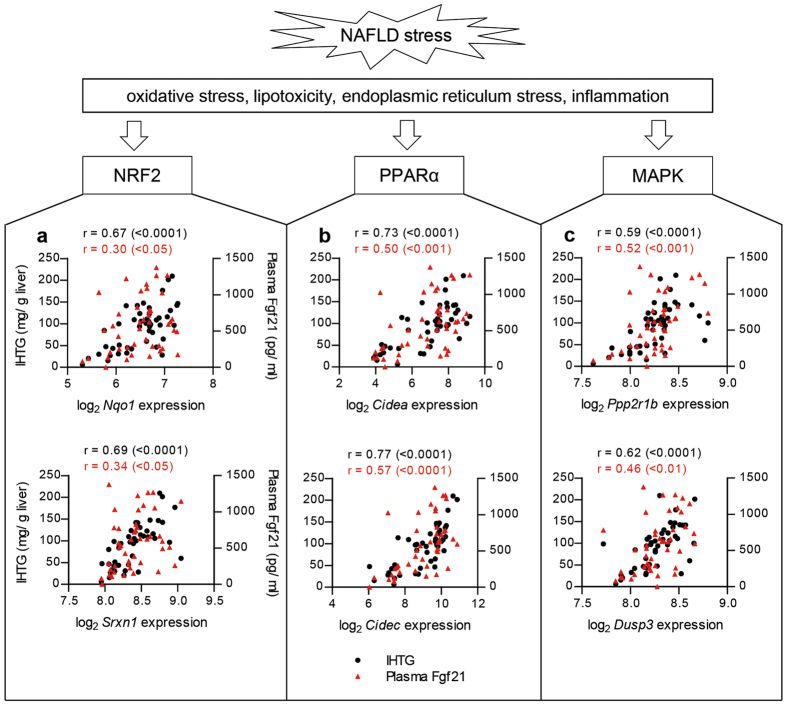
The differentially regulated pathways in NAFLD were reflected by plasma Fgf21 levels. (**a**) Stress induced by NAFLD may activate NRF2, PPARα and MAPK, and the expression of their target genes. The expression levels of the NRF2 (**b**) PPARα (**c**) and MAPK (**d**) target genes were strongly correlated with IHTG and more modestly with plasma Fgf21 levels (in black and red color, respectively). r values were calculated with Pearson’s correlations and their significance are indicated in the parentheses.

Next, to determine the relevance of Fgf21 in the NAFLD-related pathways, we first identified the pathways differentially regulated in the animals with NAFLD by performing GSEA. Then, we explored the differentially regulated pathways in NAFLD for their link to Fgf21. The gene expression data of 12, 24, and 28-month old animals were analysed with the exclusion of the CR group, since the latter group has a markedly different gene expression profile^19^. Forty-five animals were included in the microarray analysis (32 and 13 animals, with and without NAFLD, respectively). The GSEA results (based on FDR q value < 0.01), presented in Tables 3 and 4, revealed that 18 and 12 pathways were up- and down-regulated in NAFLD, respectively (lists of pathways with FDR q value < 0.05 is available in Supplementary Tables S4 and S5). The up-regulated pathways were dominated by pathways related to oxidative stress (nuclear factor (erythroid-derived 2)-like 2 or NRF2 targets, glutathione metabolism), energy and lipid metabolism (PPARα targets, oxidative phosphorylation and electron transport chain and fatty acid metabolism). The down-regulated pathways included various complement cascades pathways. Interestingly, NRF2 and PPARα targets, the 2 most significantly enriched up-regulated pathways, contained genes that have been previously identified for their strong correlation with IHTG^35^: NAD(P)H dehydrogenase quinone 1 (*Nqo1*), sulfiredoxin 1 (*Srxn1*), cell death-inducing DFFA-like effector a (*Cidea*) and c (*Cidec*). Genes in the core enrichment of NRF2 and PPARα targets are listed in Supplementary Tables S6 and S7. The gene expression levels of these genes were analysed for their correlation with IHTG, which revealed highly significant correlations (p < 0.0001; Fig. 5a,b). Although not as strong, the expression levels of these genes were also significantly correlated with the plasma Fgf21 levels. Thus, the differentially regulated PPARα and NRF2 target genes were pointed out to be the link between plasma Fgf21 levels and IHTG content during NAFLD.

Notably, MAPK targets was also among the pathways enriched in the NAFLD-differentially regulated pathway (full list of genes in core enrichment of MAPK targets in Supplementary Table S8). This is in line with the finding of MAP4K4 activation in Fig. 4d.. The top MAPK target genes, protein phosphatase 2, regulatory subunit A, beta (*Ppp2r1b*) and dual specificity phosphatase 3 (*Dusp3*), showed significant correlations to both IHTG content and plasma Fgf21 levels (Fig. 5c). Therefore, this signifies the association of plasma Fgf21 with liver cancer-related signalling.

### Gene expression response to PPARα activation demonstrates its dysregulation in NAFLD

**Figure 6:**
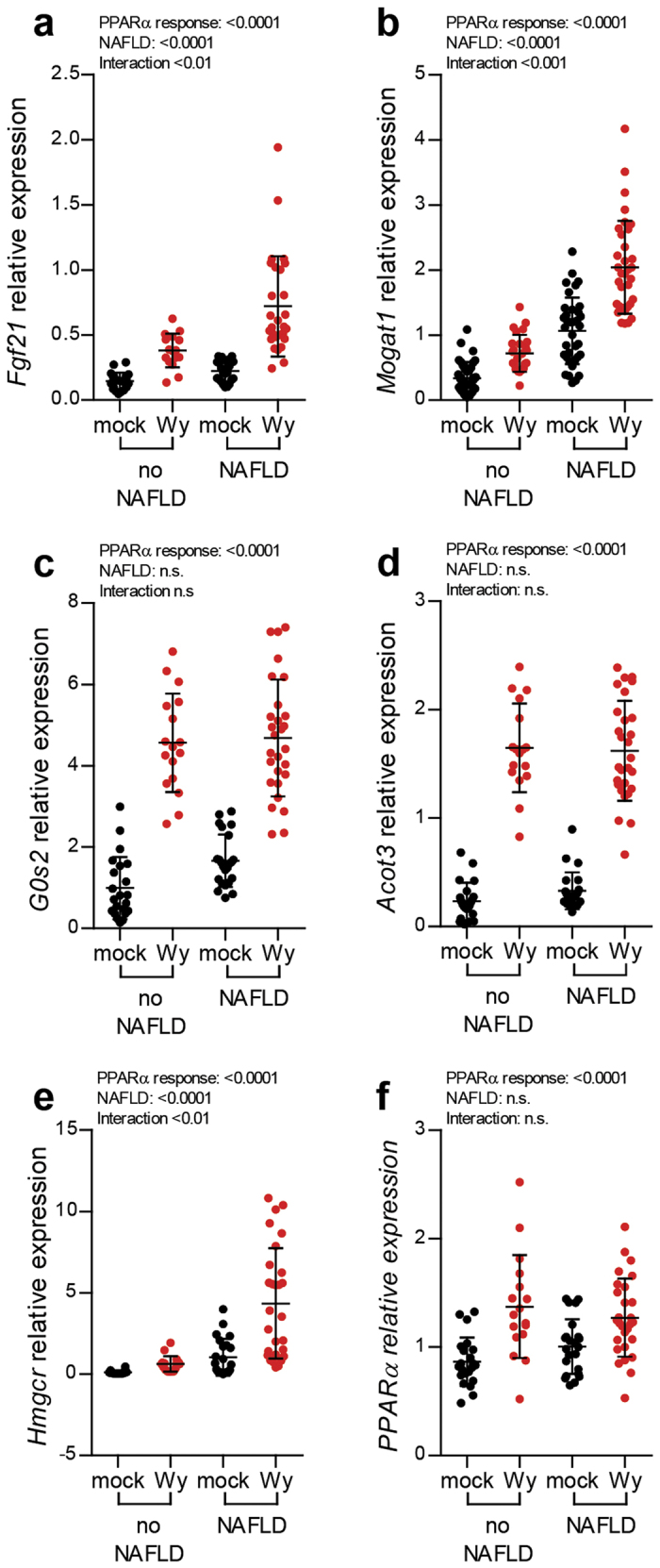
The response to PPARα challenge test showed that the presence of NAFLD partially altered the response at the gene expression levels. (**a**) *Fgf21*; (**b**) *Mogat1*; (**c**) *G0s2*; (**d**) *Acot3*; (**e**) *Hmgcr*; and (**f**) *Pparα*. The effect of PPARα agonist and NAFLD, as well as any interaction between them, were analysed using two-way ANOVA.

Based on the essential role of PPARα in lipid homeostasis, we further investigated whether the dysregulation of PPARα in NAFLD extends to an altered response when the system is challenged. To examine the response of PPARα, prior to each sacrifice the PPARα agonist Wy-14,643 (Wy) substance was administered to half of the mice of each intervention group, while the other half of the group received mock treatment. A number of PPARα target genes, including *Fgf21*^36^,^37^, were analysed by Q-PCR, namely monoacylglycerol O-acyltransferase 1 (*Mogat1*), G0/G1 switch 2 (*G0s2*), acyl-CoA thioesterase 3 (*Acot3*), hydroxymethyl glutary coenzyme A reductase (*Hmgcr*) (Fig. 6a–e). These genes were selected to represent different functions regulated by PPARα (lipogenesis: *Mogat1*; lipolysis: *G0s2*; fatty acid oxidation: *Acot3*; cholesterol metabolism: *Hmgcr*), As shown in Fig. 6a, Wy treatment led to induced hepatic *Fgf21* expression and the 2-way ANOVA test indicated an interaction between NAFLD and the Wy response. Intriguingly, animals with NAFLD exhibited an augmented response to the treatment. Similarly to *Fgf21* expression, the induction of *Mogat1* and *Hmgcr* expression levels were stronger in the animals with NAFLD (Fig. 6b,e). However, not all genes demonstrated the stochastic response to Wy treatment. *G0s2* and *Acot3* expression levels were similarly up-regulated regardless of the presence of NAFLD (Fig. 6c,d). The expression of *Pparα* itself was also examined and the results in Fig. 6f show that the stimulation of PPARα did not differ between the animals with and without NAFLD. Although NAFLD presence did not alter the response to Wy treatment of all PPARα target genes, the expression profiles of *Fgf21, Mogat1* and *Hmgcr* underscore the dysregulation PPARα signalling pathway in NAFLD.

## Discussion

In our mouse aging cohort, NAFLD development started at middle-age in Control (C)- and medium-fat (MF)-exposed mice, but not in the calorie restricted (CR)-fed animals, which stayed lean over time. The prevalence of NAFLD in the *ad libitum* C- and MF-fed groups increased during aging, which reflects weight gain and aging as risk factors for developing NAFLD^38^. In this study, we applied a long-term exposure to a less extreme diet compared to previous studies^16^,^17^ by using a 25E% medium-fat diet to mimic the slow onset of NAFLD in the human population, which did not induce severe NAFLD. We assessed this by analysing the liver fibrosis marker, 4-hydroxyproline content, and observed a lower prevalence of liver fibrosis, compared to liver steatosis or benign NAFLD.

The analysis of plasma Fgf21 levels in the different intervention groups and ages suggests that there is an age-effect on plasma Fgf21 levels, although we did not observe a diet-dependent effect on plasma Fgf21 levels. We found that at the age of 6 months, some of the mice displayed a high level of plasma Fgf21 in the absence of elevated IHTG levels. A previous study in children has also revealed a lack of correlation between serum FGF21 and NAFLD parameters^39^, moreover another study showed an inverse correlation between FGF21 and hepatic damage^40^. The addition of serum FGF21 in a NAFLD diagnostic model for children and adolescent also failed to improve the diagnostic performance^32^. An age of 6 months in mice is equivalent to mature adult age (~30 years old in human)^41^ and does not correspond to a developmental period in childhood. However, both mice and human data support either an absence of correlation or different correlation between plasma Fgf21 and NAFLD in younger age groups. Overall, our results reveal that, with the exception of the 6-month-old animals, plasma Fgf21 levels significantly correlated with IHTG content and performed well as a plasma marker for NAFLD diagnosis. This is in agreement with the results of previous studies in humans^10^,^11^.

The pathogenesis of NAFLD is attributed to a multi-hit process that includes lipotoxicity, oxidative stress and endoplasmic reticulum (ER) stress. Liver fat accumulation involves excess fatty acid supply to the liver, which triggers fatty acid oxidation and, consequently, oxidative stress from microsomal enzymes and ER stress. We found that NRF2 and PPARα targets were the most significantly enriched up-regulated pathways in the animals with NAFLD. NRF2 acts as a protective measure against oxidative stress, by producing antioxidant proteins. Meanwhile, PPARα activation is crucial for maintaining the homeostasis of fatty acid metabolism by increasing mitochondrial β-oxidation, thereby reducing the potential for fatty acid-induced lipotoxicity^42^. In addition, the WGCNA followed by pathway analysis pointed XBP1 out as one of the predicted upstream regulators of *Fgf21*. The elevated expression of FGF21 has been described as a counteractive mechanism for ER stress, by modulating lipid metabolism^43^,^44^,^45^. Therefore, the up-regulation of Fgf21 in NAFLD appears to be a simultaneous protection against lipotoxicity, oxidative stress and ER stress in NAFLD.

Intriguingly, we found that plasma Fgf21 levels at young age were related to fatty acid oxidation (L-carnitine shuttle and β-oxidation), which also corresponds to PPARα activation. While this seems to be contradictory with the idea of PPARα activation as a protective measure for fatty liver, both mouse and human studies have reported that the increased mitochondrial activity and β-oxidation do not necessarily reflect an efficient electron transport chain^46^,^47^,^48^. The authors found that the electron transport chain in subjects with hepatic steatosis and/or obesity is inefficient^46^,^47^,^48^. Therefore, it is worthwhile noting that, despite the up-regulated fatty acid oxidation in both young animals without NAFLD and old animals with NAFLD, the up-regulation in old animals with NAFLD measurement might lead to perturbing consequences, such as hepatic oxidative stress.

The *in vivo* PPARα challenge performed in this study provides a novel insight into the ability of maintaining metabolic homeostasis. A dynamic measurement by using system perturbation or challenge tests are likely more valuable to define metabolic health or resilience, compared to more static measurements^49^. By performing the PPARα agonist treatment, we demonstrate that the PPARα response at the transcriptional level is partially altered in the presence of NAFLD. It appears that liver fat is the burden of the liver’s plasticity of lipid metabolism. A similar notion was reported by Hyotylainen and co-workers, showing that high liver fat markedly hampers the ability of the liver to adaptively regulate metabolism to meet the excessive demands on basic liver functions. As a consequence, individuals with NAFLD may be more vulnerable to various metabolic stressors on the liver^50^. This underlines that, although hepatic steatosis is considered benign (first hit), when the metabolic system faces a challenge (second/multiple hits), the ability to maintain or regain homeostasis might have been compromised.

Furthermore, we revealed the association between plasma Fgf21 and transcriptional changes related to hepatocellular carcinoma (HCC) development. The predicted regulators (HNF1A, HNF4A, RICTOR, MAP4K4), functional pathways and genes (Wnt target genes *Ccnd1* and *Glul*) associated with plasma Fgf21 suggest dysregulations of metabolic and proliferative pathways^51^,^52^, which are characteristics of a benign hepatocellular tumor. Although these dysregulations alone were not sufficient to induce carcinogenesis, it likely increases the susceptibility to HCC development. In line with this hypothesis, a pre-malignant stage in NAFLD has been shown to denote stress, inflammation and even apoptosis, which pre-condition and initiate pro-oncogenic signals^53^. This is supported by the evidence that both liver expression and circulating levels of FGF21 are increased in patients with hepatitis, cirrhosis and hepatocarcinoma^54^. Therefore, the population would likely benefit from the use of plasma Fgf21 as a biomarker of an early stage of NAFLD.

Although the association between plasma Fgf21 and HCC-related function is not as strong as its performance in reflecting the benign NAFLD stage, plasma Fgf21 might be beneficial in improving the performance of biomarkers for advanced stages of NAFLD. This has been suggested in the study of a NASH biomarker, that for the purpose of defining the stages of NASH, plasma CK-18 performs better than FGF21, but adding FGF21 to the CK-18 model significantly improved the performance^14^. We evaluated the combined analysis of plasma Fgf21 and CK-18 to detect NAFLD in our study, but it did not result in a higher accuracy compared to merely Fgf21 analysis. A plausible explanation for this observation is that our mice cohort modelled a rather mild NAFLD development, so that the addition of plasma CK-18, which represents advanced stages of NAFLD, did not effectively improve the analysis.

In this study, we discovered that the expression level of *Klb*, a critical co-receptor of the FGF receptors, was negatively correlated with plasma Fgf21, IHTG and body weight. In addition, the liver expression levels of the FGF receptors *Fgfr2* and *Fgfr4* showed similar patterns, although the declining expression levels were not as strong as observed for *Klb*. FGFR2 and FGFR4 proteins, along with FGFR1, have been shown to form a transmembrane complex with β-klotho to mediate the effects of FGF21 in adipocytes^55^. Although the type of FGF receptor that forms a complex with β-klotho protein in the liver is still unclear, this observation underlines the growing notion that metabolic system might develop a resistance to mediate the beneficial effect of Fgf21. The Fgf21 resistance due to its co-receptor alteration is an essential issue to be addressed during the further development of FGF21 as a novel pharmacological agent for metabolic diseases^56^. Both human and mice studies have reported increased FGF21 gene expression or circulating protein levels with obesity and/or metabolic syndrome^57^,^58^,^59^. One of these studies also demonstrated that the diet-induced obese mice with an elevated endogenous level of Fgf21 responded poorly to acute exogenous Fgf21 administration^59^. Since *Klb* plays a critical role in mediating Fgf21’s metabolic activity^34^,^60^, the declining *Klb* expression that occurs over a long-term obesity and/or hepatic steatosis development might result in Fgf21 resistance. Therefore, in order to assess the possibility of resistance to FGF21 treatment, the consequence of the down-regulation of critical FGF21 receptors/co-receptors in the liver on sensitivity to endo- and exogenous FGF21 warrants further investigation. It is worthwhile noting that, in the acute induction of obesity in mouse model, *Klb* expression was not altered in the obese state^59^.

Taken together, in this study, we demonstrate that plasma Fgf21 levels strongly reflects liver fat accumulation, confirming its potential as NAFLD marker. However, this association is age-dependent and does not apply at the age of 6 months in the C57BL/6J mice. The molecular link between plasma Fgf21 and IHTG levels was associated with dysregulation of both metabolic and cancer-related pathways. The up-regulated Fgf21 levels in NAFLD appears to be a measure to maintain homeostasis against the adverse effects in NAFLD, e.g. lipotoxicity, oxidative stress and endoplasmic reticulum stress. The elevated plasma Fgf21 is also associated with declining expression of *Klb*, its crucial co-receptor, which suggests a resistance to Fgf21. Therefore, although liver fat accumulation is a benign stage of NAFLD, the liver is likely more vulnerable to metabolic stressors and progress to end-stage liver disease. The *in vivo* PPARα challenge further demonstrates the dysregulation of PPARα signalling in the presence of NAFLD, which results in a stochastically increasing hepatic expression of *Fgf21*. In conclusion, Fgf21 plasma levels reflect liver fat accumulation and dysregulation of metabolic pathways at a transcriptional level in the liver of C57BL/6J mice.

## Additional Information

**How to cite this article**: Rusli, F. *et al*. Fibroblast growth factor 21 reflects liver fat accumulation and dysregulation of signalling pathways in the liver of C57BL/6J mice. *Sci. Rep*. **6**, 30484; doi: 10.1038/srep30484 (2016).

## Supplementary Material

Supplementary Information

## Figures and Tables

**Table 1:** Overall performance of plasma Fgf21 levels for the diagnosis of NAFLD.

AUROC	Sensitivity (%)	Specificity (%)	PPV (%)	NPV (%)	Accuracy (%)
All time points					
0.77 (0.67–0.87)	91.4	57.1	60.4	90.3	71.4
Without 6 months old					
0.84 (0.74–0.94)	88.6	73.5	77.5	86.2	81.2

**Table 2:** NAFLD diagnosis performance of plasma Fgf21 in combination with other markers.

Fgf21 in combination with	Sensitivity (%)	Specificity (%)	PPV (%)	NPV (%)	Accuracy (%)
Plasma ALT	80.0	97.1	96.6	82.5	88.4
Plasma CK-18	82.9	79.4	80.6	81.8	81.2
Body weight	80.0	94.1	93.3	82.1	87.0

**Table 3:** List of the significantly enriched up-regulated pathways in NAFLD.

Enriched up-regulated pathways	NES*	FDR q-value
NRF2 TARGETS	2.617	0.00000
PPARA TARGETS	2.404	0.00000
WP1248 OXIDATIVE PHOSPHORYLATION	2.328	0.00000
WP295 ELECTRON TRANSPORT CHAIN	2.299	0.00068
KEGG OXIDATIVE PHOSPHORYLATION	2.260	0.00081
KEGG LYSOSOME	2.223	0.00090
WP1269 FATTY ACID BETA OXIDATION	2.208	0.00097
KEGG FATTY ACID ELONGATION	2.180	0.00135
KEGG FATTY ACID DEGRADATION	2.146	0.00210
MITOCHONDRIAL TRANSLATION	2.113	0.00337
MITOCHONDRIAL TRANSLATION TERMINATION	2.102	0.00344
MAPK TARGETS NUCLEAR EVENTS MEDIATED BY MAP KINASES	2.079	0.00418
KEGG GLUTATHIONE METABOLISM	2.052	0.00625
RESPIRATORY ELECTRON TRANSPORT ATP SYNTHESIS BY CHEMIOSMOTIC COUPLING AND HEAT PRODUCTION BY UNCOUPLING PROTEINS	2.048	0.00629
SPHINGOLIPID METABOLISM	2.032	0.00775
AQUAPORIN MEDIATED TRANSPORT	2.025	0.00786
KEGG SYNAPTIC VESICLE CYCLE	2.020	0.00795
MITOCHONDRIAL TRANSLATION INITIATION	2.003	0.00937

^*^Normalised enrichment score (NES); a statistical test for gene set enrichment.

**Table 4:** List of the significantly enriched down-regulated pathways in NAFLD.

Enriched down-regulated pathways	NES	FDR q-value
WP449 COMPLEMENT AND COAGULATION CASCADES	−2.496	0.00000
KEGG COMPLEMENT AND COAGULATION CASCADES	−2.496	0.00000
KEGG SELENOCOMPOUND METABOLISM	−2.229	0.00018
FORMATION OF FIBRIN CLOT CLOTTING CASCADE	−2.272	0.00024
WP200 COMPLEMENT ACTIVATION CLASSICAL PATHWAY	−2.219	0.00028
BIOC INTRINSICPATHWAY	−2.081	0.00213
COMMON PATHWAY	2.086	0.00226
REGULATION OF COMPLEMENT CASCADE	−2.066	0.00304
COMPLEMENT CASCADE	−2.023	0.00428
AMINO ACID TRANSPORT ACROSS THE PLASMA MEMBRANE	−1.995	0.00606
WP460 BLOOD CLOTTING CASCADE	−1.962	0.00857
INTRINSIC PATHWAY	−1.963	0.00928
