# Supplementary material for: Fibroblast growth factor 21 reflects liver fat accumulation and dysregulation of signalling pathways in the liver of C57BL/6J mice
Source: Sci Rep. 2016 Jul 29;6:30484. doi: 10.1038/srep30484 (PMC4965761; doi:10.1038/srep30484)
Supplement: Supplementary Information [file srep30484-s1.pdf]

SUPPLEMENTARY INFORMATION

Fibroblast growth factor 21 reflects liver fat accumulation and dysregulation of signalling pathways in the liver of C57BL/6J mice

Fenni Rusli[1], Joris Deelen[2], Evi Andriyani[1], Mark V Boekschoten[1], Carolien Lute[1], Erik B. van den Akker[2,3], Michael Müller[4], Marian Beekman[2], Wilma T Steegenga[1]*

[1] Nutrition, Metabolism & Genomics Group, Division of Human Nutrition, Wageningen University, 6700 EV Wageningen, The Netherlands

[2] Department of Molecular Epidemiology, Leiden University Medical Center, Leiden, The Netherlands

[3] The Delft Bioinformatics Lab, Delft University of Technology, Mekelweg 4, 2628 CD, Delft, The Netherlands

[4] Norwich Medical School, University of East Anglia, Norwich, UK

* Corresponding author. Address: Nutrition, Metabolism & Genomics Group, Division of Human Nutrition, Wageningen University, P.O. Box 8129, NL-6700 EV Wageningen, The Netherlands. Tel.: +31 317 482590, fax: +31 317 485789.

E-mail address: wilma.steegenga@wur.nl (W.T. Steegenga).

SUPPLEMENTARY TABLES

Supplementary Table S1. Composition of the experimental diet. The CR diet was adjusted for the vitamins and minerals amount to ensure a homologous intake between both groups.

	**AIN-93W**	**AIN-93W-CR**	**AIN-93W-MF**
Energy (kcal/g)	3.85	3.77	4.25
Energy from fat (%)	9	10	25
Energy from protein (%)	15	15	13
Energy from carbohydrates (%)	76	75	61
Mineral mix AIN-93M (g%)	35	50	35
Vitamin mix AIN-93M (g%)	10	14	10
Choline bitartrate (g%)	2.5	3.5	2.5

Supplementary Table S2. List of primer sequence used in Q-PCR analysis

Gene name	Forward primer (5' → 3')	Reverse primer (5' → 3')
Fgf21	GTG-TCA-AAG-CCT-CTA-GGT-TTC-TT	GGT-ACA-CAT-TGT-AAC-CGT-CCT- C
Mogat1	TCC-CGT-TGT-TCC-GAG-AAT-ATC-T	TGC-TCA-GCA-CAT-GAG-ACA-AAC
G0s2	AGT-GCT-GCC-TCT-CTT-CCC-AC	TTT-CCA-TCT-GAG-CTC-TGG-GC
Acot3	TCC-AAC-ATC-GGC-GGA-AAC-TTA	ACG-GGA-ATC-AAG-CTC-TTC-TGG
Hmgcr	AGC-TTG-CCC-GAA-TTG-TAT-GTG	TCT-GTT-GTG-AAC-CAT-GTG-ACT-TC
Pparα	TAT-TCG-GCT-GAA-GCT-GGT-GTA-C	CTG-GCA-TTT-GTT-CCG-GTT-CT
Rplp0	ATG-GGT-ACA-AGC-GCG-TCC-TG	GCC-TTG-ACC-TTT-TCA-GTA-AG

Supplementary Table S3. Characteristics of animals with/without NAFLD

	Without NAFLD (n = 53)	NAFLD (n = 36)	p-value
Body weight (g)	28.9 ± 0.9	44.5 ± 1.2	<0.0001
eWAT weight (g)	0.46 ± 0.05	1.44 ± 0.59	<0.0001
Relative eWAT weight (%)	1.43 ± 0.11	3.19 ± 0.17	<0.0001
Liver weight (g)	1.02 ± 0.04	1.71 ± 0.07	<0.0001
Relative liver weight (%)	3.50 ± 0.09	3.86 ± 0.15	0.0341
Fasting plasma insulin (ng/ml)	0.627 ± 0.093	1.767 ± 0.283	<0.0001
Plasma ALT (U/l)	6.82 ± 0.72	11.17 ± 1.03	0.0006
IHTG (mg/g liver)	22.2 ± 1.7	111.1 ± 6.2	<0.0001
Liver hydroxyproline (μg/mg liver)	0.140 ± 0.008	0.192 ± 0.015	0.0013

Data are mean ± s.e.m.; *p*-values are t-test between animals with and without NAFLD

Supplementary Table S4. List of the significantly enriched up-regulated pathways in NAFLD (FDR q-value <0.05)

Enriched up-regulated pathways	NES	FDR q-value
NRF2 TARGETS	2.617	0.00000
PPARA TARGETS	2.404	0.00000
WP1248 OXIDATIVE PHOSPHORYLATION	2.328	0.00000
WP295 ELECTRON TRANSPORT CHAIN	2.299	0.00068
KEGG OXIDATIVE PHOSPHORYLATION	2.260	0.00081
KEGG LYSOSOME	2.223	0.00090
WP1269 FATTY ACID BETA OXIDATION	2.208	0.00097
KEGG FATTY ACID ELONGATION	2.180	0.00135
KEGG FATTY ACID DEGRADATION	2.146	0.00210
MITOCHONDRIAL TRANSLATION	2.113	0.00337
MITOCHONDRIAL TRANSLATION TERMINATION	2.102	0.00344
MAPK TARGETS NUCLEAR EVENTS MEDIATED BY MAP KINASES	2.079	0.00418
KEGG GLUTATHIONE METABOLISM	2.052	0.00625
RESPIRATORY ELECTRON TRANSPORT ATP SYNTHESIS BY CHEMIOSMOTIC COUPLING AND HEAT PRODUCTION BY UNCOUPLING PROTEINS	2.048	0.00629
SPHINGOLIPID METABOLISM	2.032	0.00775
AQUAPORIN MEDIATED TRANSPORT	2.025	0.00786
KEGG SYNAPTIC VESICLE CYCLE	2.020	0.00795
MITOCHONDRIAL TRANSLATION INITIATION	2.003	0.00937
MEMBRANE TRAFFICKING	1.991	0.01022
MITOCHONDRIAL TRANSLATION ELONGATION	1.984	0.01085
BIOC MPRPATHWAY	1.975	0.01157
REGULATION OF ACTIN DYNAMICS FOR PHAGOCYTIC CUP FORMATION	1.962	0.01232
IRON UPTAKE AND TRANSPORT	1.963	0.01280
MHC CLASS II ANTIGEN PRESENTATION	1.964	0.01319
TRANSFERRIN ENDOCYTOSIS AND RECYCLING	1.943	0.01459
RESPIRATORY ELECTRON TRANSPORT	1.945	0.01479
APOPTOTIC EXECUTION PHASE	1.928	0.01634
KEGG PPAR SIGNALING PATHWAY	1.884	0.01960
PHAGOSOMAL MATURATION EARLY ENDOSOMAL STAGE	1.910	0.01961
WP2316 PPAR SIGNALING PATHWAY	1.887	0.01962
KEGG SPHINGOLIPID METABOLISM	1.887	0.02005
PROSTACYCLIN SIGNALLING THROUGH PROSTACYCLIN RECEPTOR	1.889	0.02012
CAM PATHWAY	1.890	0.02023
KEGG VASOPRESSIN REGULATED WATER REABSORPTION	1.895	0.02026
VASOPRESSIN REGULATES RENAL WATER HOMEOSTASIS VIA AQUAPORINS	1.877	0.02040
LYSOSOME VESICLE BIOGENESIS	1.896	0.02043
CALMODULIN INDUCED EVENTS	1.892	0.02051
BIOC CHREBPPATHWAY	1.898	0.02056
CA DEPENDENT EVENTS	1.900	0.02085

THE CITRIC ACID TCA CYCLE AND RESPIRATORY ELECTRON TRANSPORT	1.900	0.02152
WP401 MITOCHONDRIAL LC FATTY ACID BETA OXIDATION	1.870	0.02157
MITOTIC PROPHASE	1.859	0.02232
SYNTHESIS AND INTERCONVERSION OF NUCLEOTIDE DI AND TRIPHOSPHATES	1.860	0.02243
TRANSLOCATION OF GLUT4 TO THE PLASMA MEMBRANE	1.864	0.02248
DARPP 32 EVENTS	1.855	0.02260
NUCLEAR EVENTS KINASE AND TRANSCRIPTION FACTOR ACTIVATION	1.850	0.02261
LATENT INFECTION OF HOMO SAPIENS WITH MYCOBACTERIUM TUBERCULOSIS	1.851	0.02286
KEGG BIOSYNTHESIS OF UNSATURATED FATTY ACIDS	1.860	0.02289
INSULIN RECEPTOR RECYCLING	1.842	0.02434
KEGG AMINO SUGAR AND NUCLEOTIDE SUGAR METABOLISM	1.842	0.02471
GLUCAGON TYPE LIGAND RECEPTORS	1.833	0.02616
KEGG COLLECTING DUCT ACID SECRETION	1.828	0.02650
GLYCEROPHOSPHOLIPID BIOSYNTHESIS	1.822	0.02679
KEGG OOCYTE MEIOSIS	1.829	0.02688
APOPTOTIC CLEAVAGE OF CELLULAR PROTEINS	1.823	0.02724
CITRIC ACID CYCLE TCA CYCLE	1.813	0.02917
GLYCOSPHINGOLIPID METABOLISM	1.794	0.03413
INTRINSIC PATHWAY FOR APOPTOSIS	1.790	0.03454
KEGG ALCOHOLISM	1.779	0.03656
BIOC BIOPEPTIDESPATHWAY	1.780	0.03688
TRANS GOLGI NETWORK VESICLE BUDDING	1.781	0.03706
WP2087 MIRNA REGULATION OF DNA DAMAGE RESPONSE	1.782	0.03737
FATTY ACID TRIACYLGLYCEROL AND KETONE BODY METABOLISM	1.774	0.03805
G ALPHA Z SIGNALLING EVENTS	1.768	0.03839
WP317 GLYCOGEN METABOLISM	1.769	0.03877
KEGG PARKINSON S DISEASE	1.769	0.03938
METABOLISM OF NUCLEOTIDES	1.763	0.03947
ADP SIGNALLING THROUGH P2Y PURINOCEPTOR 12	1.759	0.04059
CLATHRIN DERIVED VESICLE BUDDING	1.752	0.04113
BIOC CREBPATHWAY	1.755	0.04113
KEGG DOPAMINERGIC SYNAPSE	1.750	0.04146
ACTIVATION OF BAD AND TRANSLOCATION TO MITOCHONDRIA	1.752	0.04172
KEGG TIGHT JUNCTION	1.746	0.04235
KEGG RETINOL METABOLISM	1.736	0.04585
SEMA3A PAK DEPENDENT AXON REPULSION	1.726	0.04880
POST CHAPERONIN TUBULIN FOLDING PATHWAY	1.727	0.04916

Supplementary Table S5. List of the significantly enriched down-regulated pathways in NAFLD (FDR q-value <0.05)

Enriched down-regulated pathways	NES	FDR q-value
WP449 COMPLEMENT AND COAGULATION CASCADES	-2.496	0.00000
KEGG COMPLEMENT AND COAGULATION CASCADES	-2.496	0.00000
KEGG SELENOCOMPOUND METABOLISM	-2.229	0.00018
FORMATION OF FIBRIN CLOT CLOTTING CASCADE	-2.272	0.00024
WP200 COMPLEMENT ACTIVATION CLASSICAL PATHWAY	-2.219	0.00028
BIOC INTRINSICPATHWAY	-2.081	0.00213
COMMON PATHWAY	-2.086	0.00226
REGULATION OF COMPLEMENT CASCADE	-2.066	0.00304
COMPLEMENT CASCADE	-2.023	0.00428
AMINO ACID TRANSPORT ACROSS THE PLASMA MEMBRANE	-1.995	0.00606
WP460 BLOOD CLOTTING CASCADE	-1.962	0.00857
INTRINSIC PATHWAY	-1.963	0.00928
GLUTAMATE NEUROTRANSMITTER RELEASE CYCLE	-1.930	0.01245
BMAL1 CLOCK NPAS2 ACTIVATES CIRCADIAN GENE EXPRESSION	-1.832	0.04132
SYNTHESIS OF BILE ACIDS AND BILE SALTS VIA 7ALPHA HYDROXYCHOLESTEROL	-1.821	0.04408
WP310 MRNA PROCESSING	-1.806	0.04612
KEGG PROTEIN EXPORT	-1.806	0.04900

Supplementary Table S6. The list of genes in core enrichment of NRF2 targets

	NRF2	Rank in gene
1	NQO1	24
2	UGDH	36
3	SRXN1	44
4	GPX1	60
5	ABCB1A	99
6	ALDH1A7	112
7	SULT1C2	128
8	GCLC	141
9	FTH1	151
10	EPHX1	164
11	GSTM3	287
12	GSTM4	332
13	ALDH3A2	344
14	PGD	347
15	SQSTM1	387
16	ALDH1A1	508
17	CES1G	689
18	GSTM1	730
19	GSTM5	767
20	CBR3	818
21	ABCC4	864
22	TKT	899
23	ALDH9A1	910
24	BLVRB	1076
25	GSTM2	1115
26	GPX3	1527
27	GSS	1726

Supplementary Table S7. The list of genes in core enrichment of PPARα targets

	PPARα	Rank in gene		PPARα	Rank in gene
1	CIDEA	0	42	HMGCL	1074
2	UGT1A9	1	43	ACADM	1096
3	CIDEC	2	44	ACOT9	1144
4	MOGAT1	9	45	ELOVL5	1147
5	PLIN4	27	46	PCTP	1178
6	CD36	35	47	CYP3A11	1220
7	ECH1	38	48	EHHADH	1251
8	CPT1B	55	49	HSD17B10	1307
9	AQP7	56	50	ACAT1	1309
10	SLC27A4	73	51	ACAA1B	1397
11	ACOT2	75	52	SLC25A20	1458
12	CYP4A14	86	53	ECI1	1490
13	PEX11A	110	54	ACOT5	1501
14	PLIN2	139	55	ACOT8	1507
15	VLDLR	173	56	ACACB	1521
16	CRAT	244	57	CPT2	1543
17	CYP4A10	306	58	GPAM	1592
18	ALDH3A2	344	59	AGPAT3	1671
19	AGXT2	360	60	HADH	1683
20	ELOVL7	410	61	ABCD2	1809
21	LIPA	428	62	CYP4A12A	1826
22	HADHA	449	63	ACOT1	1989
23	ACAD9	509	64	UCP3	2131
24	IL1RN	525	65	ACSM3	2132
25	FABP2	541	66	SCD2	2139
26	ODC1	549	67	PLTP	2292
27	SLC25A10	634	68	OAT	2367
28	CROT	687	69	CPT1A	2517
29	CES1G	689	70	ACOT7	2553
30	FGF21	699	71	ACOX1	2576
31	PDK4	713	72	CYP2J6	2601
32	LIPE	716	73	GPD2	2629
33	ACOT3	738	74	ACADL	2813
34	GYK	776	75	HSD17B4	2842
35	ALDH9A1	910	76	MGLL	2994
36	ACOT4	916	77	ETFDH	2997
37	ACAD10	928	78	ACOT10	3011
38	RAB9	1033	79	ABCB4	3036
39	DECR2	1050	80	UCP2	3040
40	DECR1	1055	81	ACAA1A	3110
41	TXNIP	1065			

Supplementary Table S8. The list of genes in core enrichment of MAPK targets

MAPK	Rank in gene list
PPP2R1B	12
DUSP3	115
MAPK3	621
JUN	1001
MAPK9	1066
MAPKAPK2	1214
RPS6KA1	1407
MAPK8	1570
MAPK10	2011
PPP2R1A	2102
PPP2CA	2797

SUPPLEMENTARY FIGURES

Supplementary Figure S1

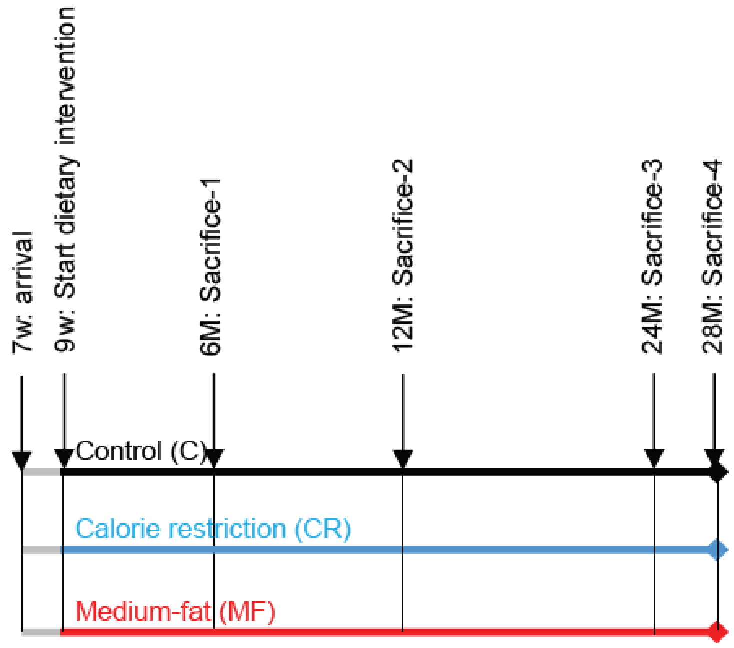

Supplementary Figure S1. Study design scheme. The male C57BL/6J mice arrived at 7 weeks old and were acclimatized for 2 weeks. The dietary intervention was started at the age of 9 weeks and the mice were culled at 6, 12, 24 and 28 months, in order to cover different life stages.

Supplementary Figure S2

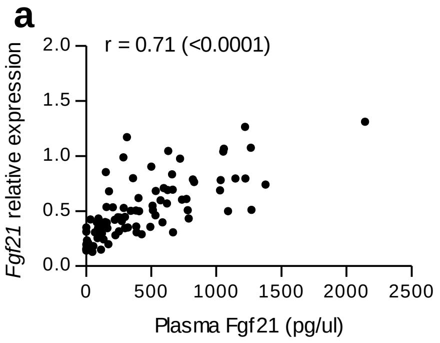

Supplementary Figure S2a. Validating the microarray data, plasma Fgf21 levels were positively correlated with hepatic Fgf21 expressions, which was obtained through Q-PCR technique. r value and its significance were calculated with Pearson's correlation.

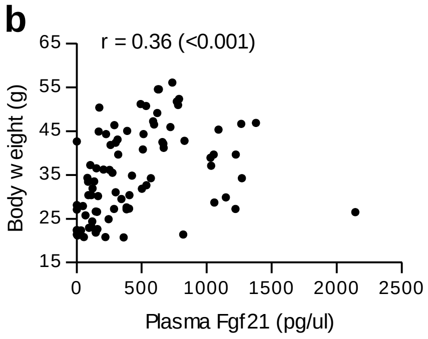

Supplementary Figure S2b. Plasma Fgf21 levels were positively correlated with body weight. r value and its significance were calculated with Pearson's correlation.

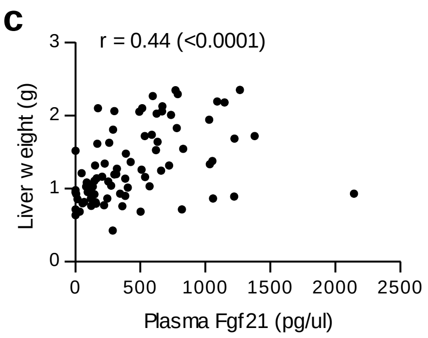

Supplementary Figure S2c. Plasma Fgf21 levels were positively correlated with liver weight. r value and its significance were calculated with Pearson's correlation.

Supplementary Figure S3

a

ROC curve for all time points

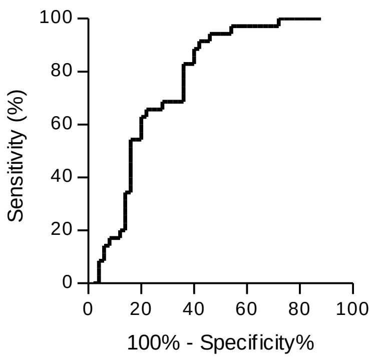

Supplementary Figure S3a. ROC analysis of plasma Fgf21 predicting animals with and without NAFLD. In this analysis, all animals from 4 age time points were included.

b

ROC curve for 12, 24, 28M

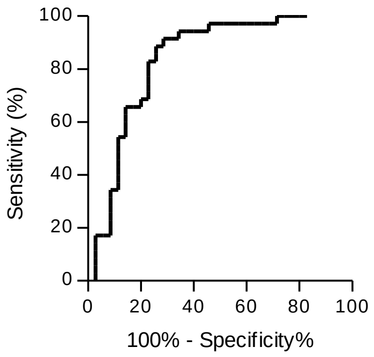

Supplementary Figure S3b. ROC analysis of plasma Fgf21 predicting animals with and without NAFLD at middle and old age. The 6 month-old mice were excluded in this analysis.

Supplementary Figure S4

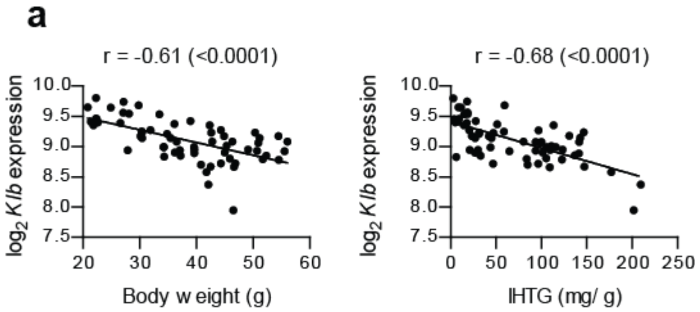

Supplementary Figure S4a. Body weight and intrahepatic triglyceride (IHTG) were inversely correlated with *Klb* expression levels. r values and their significance (in parentheses) were calculated with Pearson’s correlation.

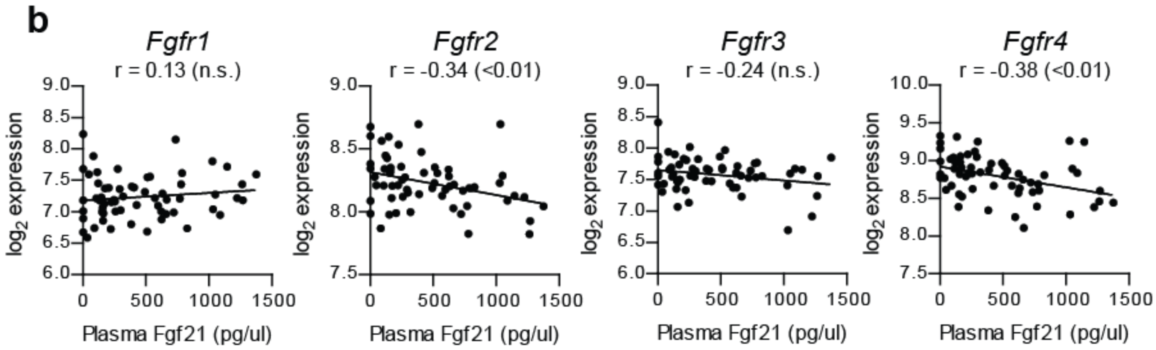

Supplementary Figure S4b. Correlations between the plasma Fgf21 and gene expression levels of *Fgfr1, Fgfr2, Fgfr3,* and *Fgfr4.* r values and their significance (in parentheses) were calculated with Pearson’s correlation.

Supplementary Figure S5

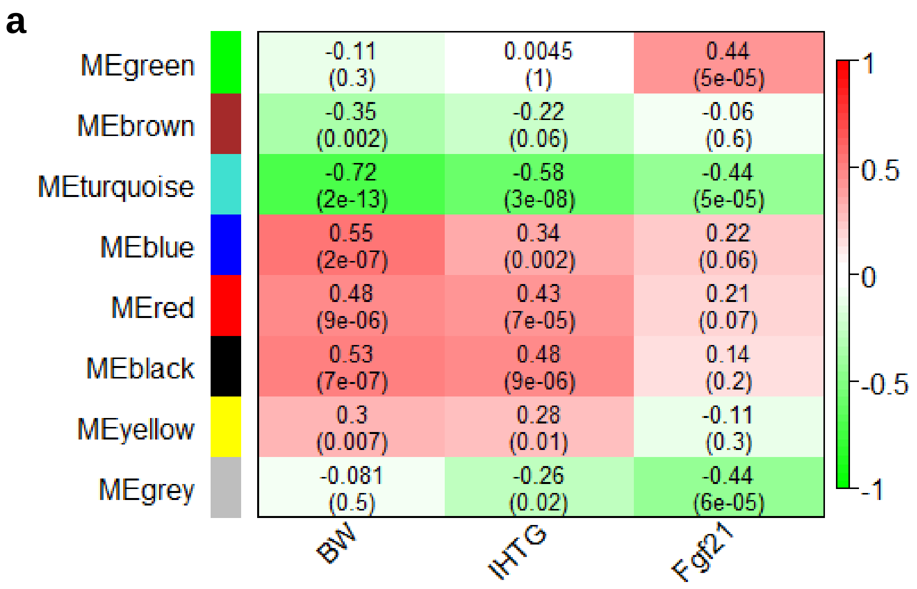

Supplementary Figure S5a. Heat map depicting the correlation between gene modules (in rows) and phenotypes (in columns). To investigate the functions of the genes associated with the elevated Fgf21 plasma level at young age without accumulation of IHTG, this analysis was performed with the inclusion of the 6 month old animals. In this analysis, the variable deepSplit setting in WGCNA was fine-tuned to obtain a module with a strong correlation with plasma Fgf21 levels, but not with IHTG content. The top values in each cell represents the correlation coefficient between the module and phenotype with the correlation p-value in parentheses. Red and green color represents positive and negative correlation, respectively.

b

**IPA results**	**Biological processes/regulators**	***p*-value/ z-score**
Liver-specific functions	Liver steatosis<br>Hepatocellular peroxisome proliferation<br>Liver damage	2.39×10^{-1} – 4.18×10^{-3}<br>4.28×10^{-2} – 1.36×10^{-8}<br>5.51×10^{-1} – 1.15×10^{-3}
Canonical pathways	Mitochondrial L-carnitine shuttle pathway<br>Fatty acid β-oxidation I<br>Stearate biosynthesis I (animals)	8.09×10^{-7}<br>1.11×10^{-6}<br>2.29×10^{-6}
Upstream regulators	PPARα<br>PPARGC1α<br>PPARγ	5.905<br>3.349<br>3.313

Supplementary Figure S5b. Biological processes, pathways and regulators associated with MEgreen (in Supplementary Figure S5a). Significant liver-specific functions and canonical pathways are reported in p-values. Significant upstream regulators are reported in predicted activation z-score. Positive and negative z-score represent predicted activation and inhibition, respectively.
